# Downscaling the Analysis of Complex Transmembrane Signaling Cascades to Closed Attoliter Volumes

**DOI:** 10.1371/journal.pone.0070929

**Published:** 2013-08-05

**Authors:** Luigino Grasso, Romain Wyss, Joachim Piguet, Michael Werner, Ghérici Hassaïne, Ruud Hovius, Horst Vogel

**Affiliations:** Laboratory of Physical Chemistry of Polymers and Membranes, Ecole Polytechnique Fédérale de Lausanne, Lausanne, Switzerland; University of Oldenburg, Germany

## Abstract

Cellular signaling is classically investigated by measuring optical or electrical properties of single or populations of living cells. Here we show that ligand binding to cell surface receptors and subsequent activation of signaling cascades can be monitored in single, (sub-)micrometer sized native vesicles with single-molecule sensitivity. The vesicles are derived from live mammalian cells using chemicals or optical tweezers. They comprise parts of a cell’s plasma membrane and cytosol and represent the smallest autonomous containers performing cellular signaling reactions thus functioning like minimized cells. Using fluorescence microscopies, we measured in individual vesicles the different steps of G-protein-coupled receptor mediated signaling like ligand binding to receptors, subsequent G-protein activation and finally arrestin translocation indicating receptor deactivation. Observing cellular signaling reactions in individual vesicles opens the door for downscaling bioanalysis of cellular functions to the attoliter range, multiplexing single cell analysis, and investigating receptor mediated signaling in multiarray format.

## Introduction

Miniaturized bioassays of cellular signaling are of fundamental importance to increase both throughput and number of parameters evaluated, and substantially decrease sample consumption. Microfluidics and microarray technologies are currently used in this context to monitor cellular signaling in a highly parallelized and automated fashion typically in nano- to picoliter volumes [1], [2]. Downscaling to smaller volumes using intact mammalian cells is not feasible with such approaches. As outlined elsewhere [3]–[5], a widely overlooked large potential for miniaturization of bioanalysis and for nano-biotechnology lies in single, closed (sub-)femtoliter reaction volumes isolated from the ensemble and investigated as individuals, by micromanipulation, by self-positioning in microarrays on surfaces or in multiple optical traps free in solution [6], [7]. Although micrometer-sized lipid vesicles or polymeric containers have been used to observe simple biochemical reactions [8]–[12], the reliable reconstitution of complex transmembrane cellular signaling cascades into such artificial containers has never been shown and seems to be difficult to realize for the near future. In this context, plasma membrane vesicles, derived from living mammalian cells by chemical treatment [3], [13], [14] or opto-mechanical manipulation [15] are of utmost interest. As a native vesicle receives from its mother cell a portion of naturally oriented plasma membrane and part of the cytoplasm, it should be regarded as a single-cell biopsy, able to act as a miniaturized, minimal autonomous entity detecting external signals at and transmitting them across the vesicle plasma membrane, and finally activating signaling reaction cascades inside the vesicle similar to its mother cell.

Up until now cell-derived plasma membrane vesicles have been used mostly as model membranes to study the lateral distribution of lipids and proteins within the plasma membrane of individual vesicles using optical microscopy [16]–[18] as well as the chemical composition of vesicle populations using mass spectrometry [19]. Only a few studies addressed the function of signaling receptor proteins in the plasma membrane of individual native vesicles such as binding of agonists to and activating of ligand-gated ion channels [3] or ligand binding to G-protein-coupled receptors (GPCRs) [15]. Transmembrane receptor mediated signaling reactions in native vesicles have yet not been demonstrated.

Here, we present first results of detecting complex signaling pathways in individual vesicles focusing on G-protein-coupled receptors as an example of central importance for transmembrane cellular signaling cascades [20]. We demonstrate the ability of single cell-derived plasma membrane vesicles to convert an external stimulus to an internal response by monitoring the different steps of GPCR mediated signaling, from initial signal detection (ligand binding) to subsequent transmission of the external signal across the vesicle’s plasma membrane leading to intravesicular signaling reactions (activation of G-proteins) and finally receptor deactivation (arrestin translocation). We derived plasma membrane vesicles from live mammalian cells either by incubating the cells with cytochalasin B, a compound known to destabilize the interaction between the actin cytoskeleton and the plasma membrane, or by micromechanical single-cell biopsy using an optical tweezer. As shown below, both sorts of vesicles reveal similar properties concerning composition and functional signaling cascades.

## Results and Discussion

### Receptor Density and Diffusion

**Figure 1:**
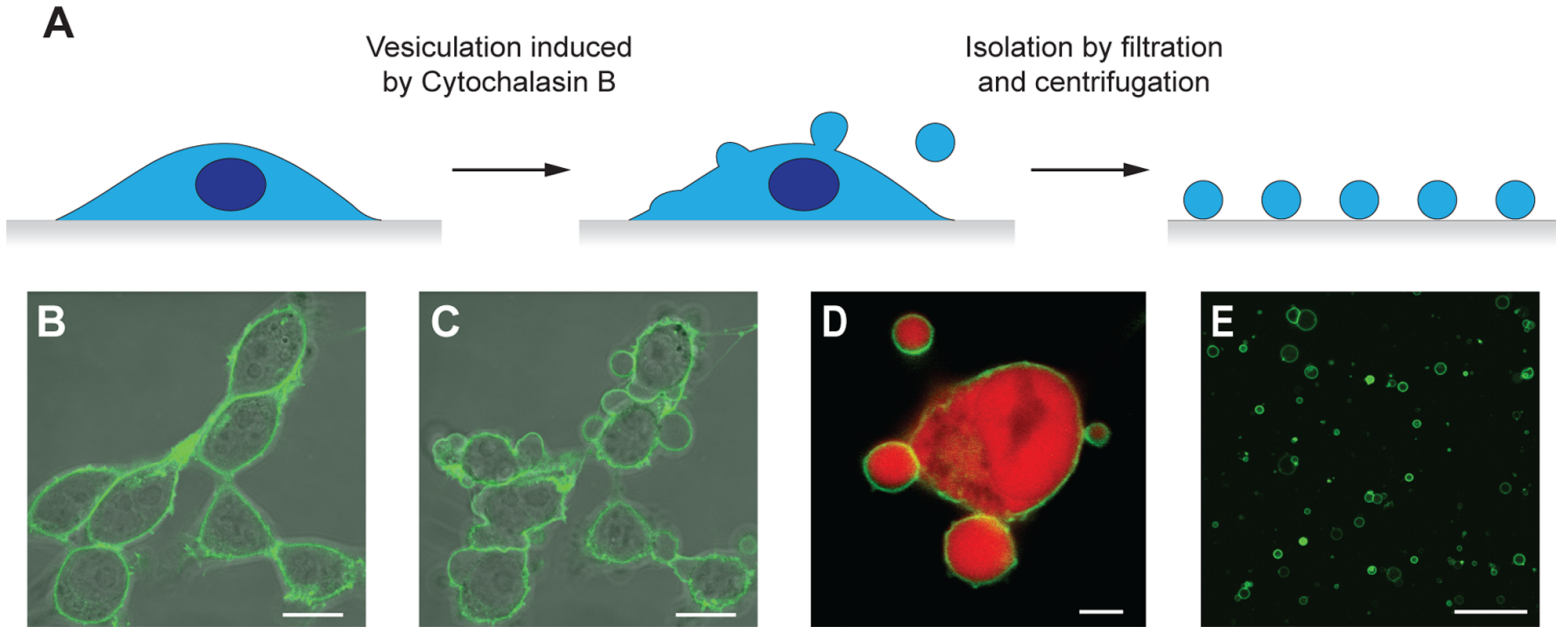
Formation of plasma membrane vesicles from live cells. (A) Scheme: After adding cytochalasin B, cultured cells formed within a few minutes blebbing structures on their plasma membranes which can be sheared off (by shaking or by pulling with an optical tweezer) as (sub-)micrometer-sized closed plasma membrane vesicles. (B,C) Confocal micrographs showing the YFP fluorescence of HEK cells expressing A2AR-YFP before (B) and after (C) addition of cytochalasin B (typical final concentration 25 µg/ml); scale bars: 10 µm. (D) Confocal micrograph of a blebbing cell expressing a fluorescent membrane receptor (A2A-YFP, green) and a cytosolic protein (mCherry, red). Both proteins are present in the cell and in the shed vesicles; scale bar: 3 µm. (E) Array of vesicles produced from HEK cells expressing A2AR-YFP; scale bar: 10 µm.

**Figure 2:**
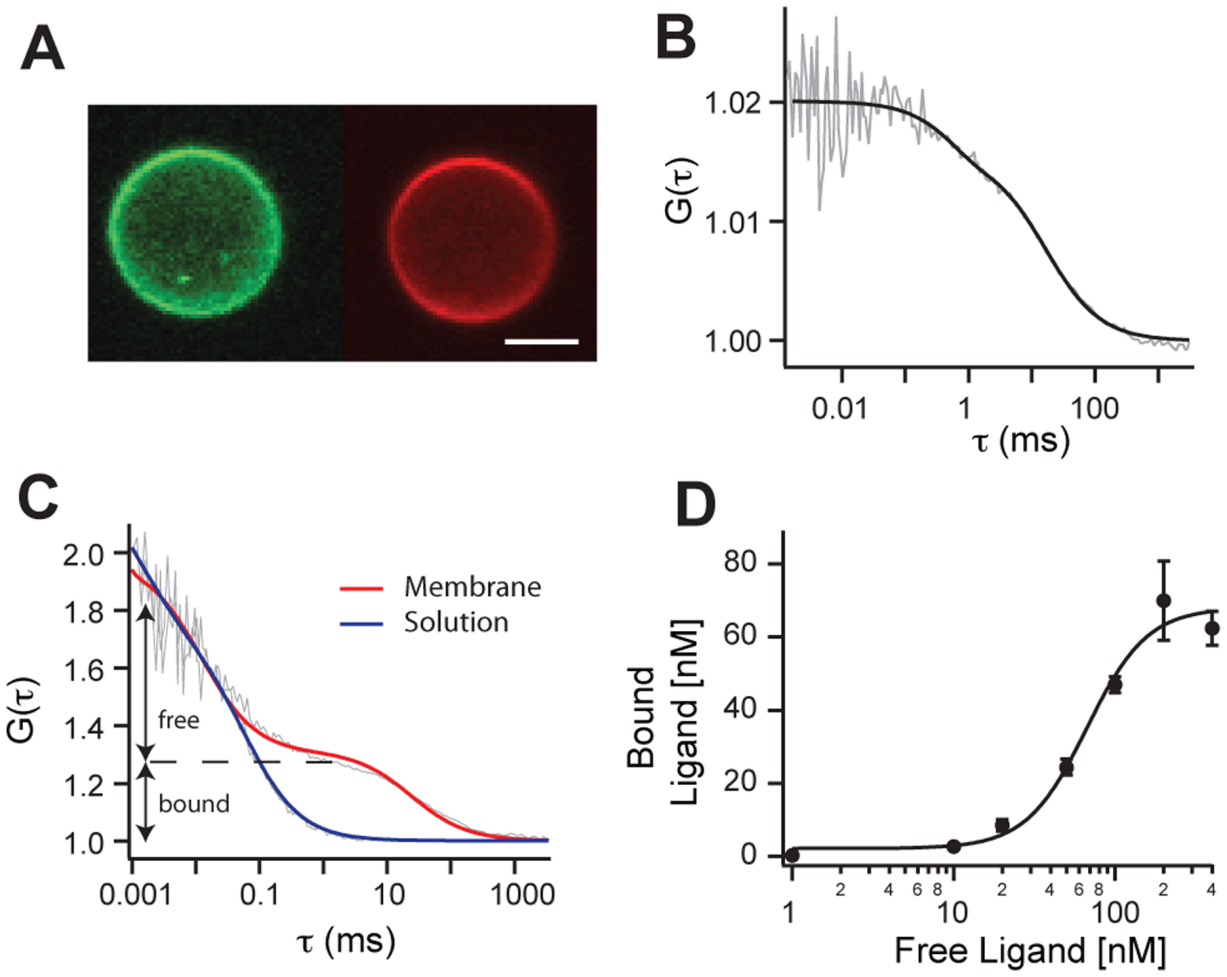
Diffusion of and ligand binding to GPCR. (A) Confocal micrograph of a plasma membrane vesicle derived from a HEK cell expressing heterologously the A2AR-YFP showing the fluorescence of the A2AR-YFP (green, left) and the fluorescence of the receptor-bound antagonists XAC-Atto655 (red, right); scale bar: 1 µm. Intensity fluctuations were recorded for 100s yielding to the autocorrelation curve of A2AR-YFP (B) that was best fit with two correlation times: the diffusion time of the receptor (τ*_R_* = 19.9±3.5 ms) and the chromophore blinking time (τ*_T_* = 574±289 µs). (C) Normalized autocorrelation curves of XAC-Atto655 measured in the supernatant (blue), at the apical membrane of the vesicle (red). (D) Receptor binding of XAC-Atto655 at different concentrations yielded a dissociation constant of *K_D_* = 67±11 nM. Shown are data points and standard deviations of the mean of three independent titrations performed on different individual native vesicles.

We first report on experiments using vesicles obtained by cytochalasin B treatment. The formation of plasma membrane vesicles is depicted schematically in Figure 1A and in the micrographs of Figure 1B–E for HEK cells expressing fluorescent membrane and cytosolic proteins, demonstrating that the (sub)micrometer sized vesicles (size distribution: Figure S1) comprise portions of a cell’s membrane and cytosol. Using fluorescence correlation spectroscopy (FCS), we determined the concentration and mobility of a prototypical GPCR, the adenosine A2A receptor fused to YFP (A2AR-YFP), in the plasma membrane of both individual native vesicles and their mother cells. From the measured autocorrelation function (ACF) (Figure 2B and Figures S2A,B), we calculated receptor densities of 500±41 (*n* = 21) and 580±39 (*n* = 21) receptors/µm^2^ in the plasma membranes of the vesicles and the mother cells, respectively. These data show that during the formation of plasma membrane vesicles the native receptor density is maintained. The mobility of A2AR-YFP was also investigated by FCS (Figure 2B). The measured ACF curves were best described by 2D-diffusion of a single component and considering triplet state formation, yielding a typical receptor diffusion coefficient *D* = 0.59±0.04 µm^2^/s (*n* = 21) in vesicles, and *D* = 0.17±0.02 µm^2^/s (*n* = 21) in cells (Figure S2A,B). The difference of the receptor mobility in cells and vesicles might be due to the interaction of GPCRs with the cell’s cytoskeleton [21] and local roughness of the cell’s plasma membrane, which both are absent in cytochalasin-derived vesicles [3], [22]. Plasma membrane vesicles are particularly suited to measure processes on/in membranes by FCS as vesicles do not show morphological changes, which are typical for living cells [23]. Therefore the measured fluorescence traces are very stable and reproducible with considerable lower background fluorescence as compared to cells. In contrast, FCS measurements on the plasma membranes of living cells are prone to artifacts due to the intrinsic movements of a cell.

### Ligand Binding

The first step of GPCR-mediated signaling concerns the binding of a ligand molecule to its cognate receptor. To monitor this interaction, we used XAC-Atto655 a fluorescent antagonist for A2AR [24] (Figure 2A). FCS enables to distinguish free from receptor-bound XAC-Atto655 according to their distinct different diffusion coefficients of *D_free_* = 377 µm^2^/s and *D_bound_* = 0.53 µm^2^/s (Figure 2C). The specificity of the interaction of XAC-Atto655 with A2AR was demonstrated by (i) measuring its dissociation from the receptor adding an excess of unlabeled XAC (Figure S3) and (ii) determining from ACFs obtained at various ligand concentrations a dissociation constant of *K_D_* = 67±11 nM (Figure 2D), which is close to *K_D_* = 97±12 nM we measured on cells (Figure S2C,D).

### G-protein Activation

**Figure 3:**
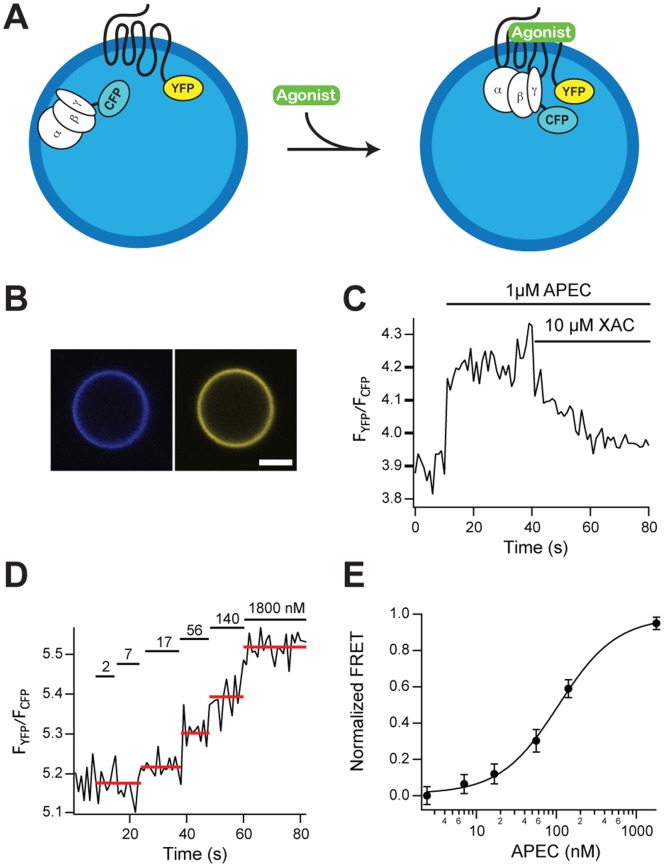
Transmembrane signaling in cell-derived plasma membrane vesicles. (A) Scheme of the activation of G-proteins inside a single native vesicle. Upon binding to an agonist A2AR-YFP receptor forms a complex with the heterotrimeric Gα_s_β_1_γ_2_-CFP measured by FRET between CFP of Gγ_2_ and YFP of A2AR. (B) Confocal micrograph of a typical plasma membrane vesicle derived from a HEK cell expressing heterologously Gγ_2_-CFP (blue, left) and A2AR-YFP (yellow, right); scale bar: 1 µm. (C) FRET is detected as fluorescence intensity ratio *F_YFP_/F_CFP_* within a single native vesicle; addition of 1 µM agonist APEC significantly increases FRET and subsequent addition of excess of antagonist XAC (10 µM) results in a decrease of the FRET signal. (D) The FRET signal increases with the concentration of agonist APEC added to the bulk solution (concentrations indicated above the bars in nM). (E) A dose-dependent increase of the FRET yields *EC_50_* = 100±11 nM; shown are averages and standard deviations of the mean of nine titration experiments each performed on different, individual vesicles.

In living cells, the subsequent interactions between GPCRs and their G-proteins have been observed by fluorescence resonance energy transfer (FRET) using fluorescent-labeled partners [25]. Here we monitor these interactions for the first time in individual, (sub-)micrometer-sized plasma membrane vesicles by measuring FRET between CFP-labeled G-proteins and A2AR-YFP (Figure 3A). Vesicles were derived from cells transiently expressing the fluorescent proteins A2AR-YFP and Gγ_2_-CFP, together with unlabeled Gα_s_ and Gβ_1_. Whereas an entire expressing cell exhibits high fluorescence located at the endomembranes due to continuous expression of the labeled proteins (Figure S4A,B), the lumen of a plasma membrane vesicle is devoid of such fluorescence (Figure 3B) as it does not carry any endoplasmic reticulum [3]. Addition of agonist significantly increases FRET (measured as fluorescence intensities ratio *F_YFP_*/*F_CFP_*), showing that the ligand-bound receptor interacts with the heterotrimeric G-protein and subsequent antagonist addition results in a reversible decrease of the FRET signal (Figure 3C and Figure S5). These results imply not only that native vesicles are sensitive to extracellular stimuli, but also that they are able to transmit the signal of ligand-binding via the receptor across the membrane towards the G-protein. Moreover, increasing agonist concentration in the bulk leads to a concomitant increase of the FRET signal saturating at high agonist concentrations with an *EC_50_* = 100±11 nM (Figure 3D,E). This value is consistent with *EC_50_* = 76±29 nM measured in cells (Figure S4C,D).

### Receptor Desensitization

**Figure 4:**
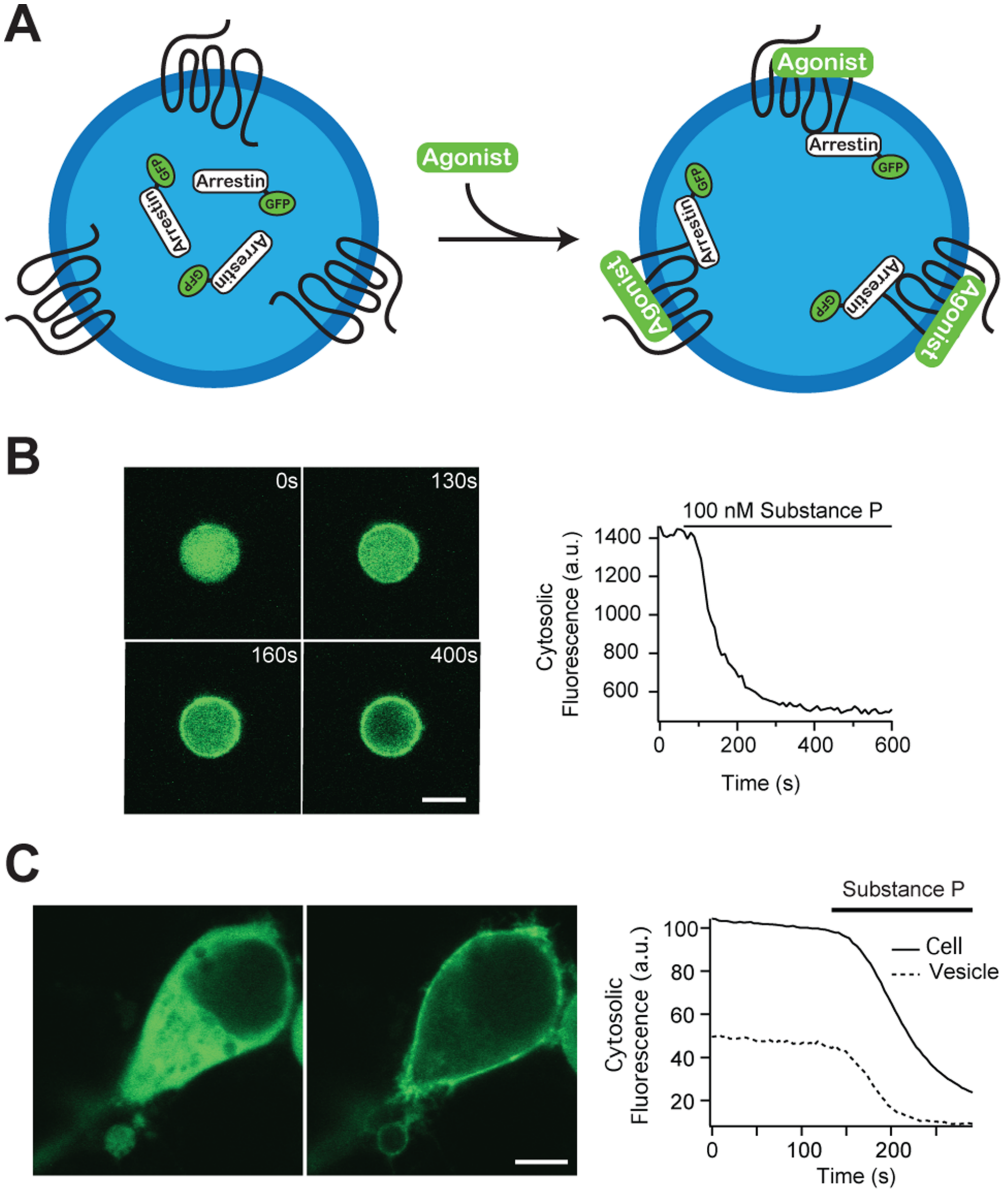
GPCR desensitization in cell-derived plasma membrane vesicles. (A) Scheme: Binding of agonists to GPCRs initiates receptor phosphorylation, which in turn leads to binding of arrestin to the GPCRs preventing further activation of G proteins. Here, we monitor by confocal microscopy in an individual native vesicle the translocation of fluorescent arrestin (arrestin-GFP) from the lumen to the membrane containing NK1R. (B) Confocal micrograph of a particular single plasma membrane vesicle showing the fluorescence of arrestin-GFP. The agonist substance P added to the bulk binds to the NK1R and induces rapid recruitment of arrestin-GFP at the vesicle membrane (scale bar: 2 µm) as shown in as a time course of the luminal fluorescence of arrestin-GFP. (C) Confocal micrographs of a plasma membrane vesicle and its mother cell expressing NK1R and arrestin-GFP, showing the fluorescence signal of arrestin-GFP before (left) and after (right) substance P perfusion (scale bar: 5 µm) and the time course of both the cytosolic fluorescence of arrestin-GFP in the cell and in the vesicle.

For many GPCRs agonist-induced activation is followed by downstream receptor deactivation. Agonist-bound GPCRs rapidly undergo selective phosphorylation by G-protein-coupled receptor kinases (GRKs) and second-messenger kinases, e.g. cAMP-dependent protein kinase (PKA) and protein kinase C (PKC). This finally leads to the binding of arrestin to the GPCRs preventing further interaction of receptors with G-proteins, thus effectively terminating the G-protein-mediated signaling [26] (Figure 4A). To investigate whether this pathway can be activated also within the vesicles, we employed the neurokinin-1 receptor (NK1R), a GPCR known to exhibit rapid arrestin-mediated desensitization [27]. GFP-tagged β-arrestin2 (arrestin-GFP) was co-expressed together with NK1R in cells used for the vesicle production. A micrograph of a typical single vesicle is depicted in Figure 4B. Upon binding of the agonist substance P to the NK1R, arrestin-GFP redistributed from the lumen to the membrane of the vesicle, demonstrating that receptor activation finally leads to the formation of the arrestin-receptor complex. Moreover, these observations imply that native vesicles contain the functional downstream signaling machinery comprising the different kinases required to induce receptor phosphorylation and desensitization. To demonstrate the identical functional behavior of arrestin in vesicles and cells, we tested the activation of arrestin in a newly formed vesicle still in the vicinity of its mother cell. Cells expressing NK1R and arrestin-GFP were treated with cytochalasin B in order to produce native vesicles (Figure 4C). When substance P was added to the bulk solution, the arrestin was simultaneously recruited to the plasma membrane of the vesicle and of the cell.

### Production and Isolation of Vesicles Using an Optical Tweezer

**Figure 5:**
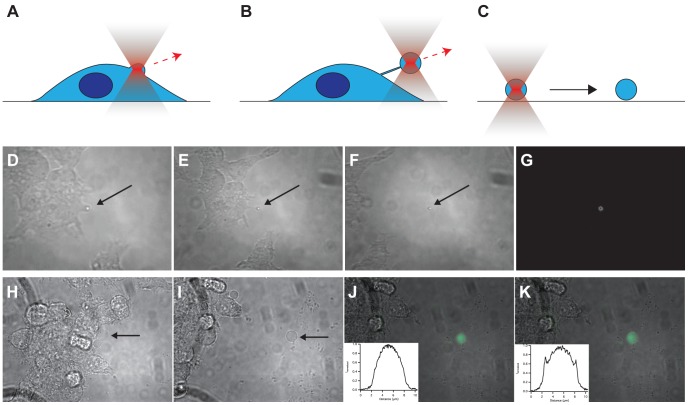
Isolation of plasma membrane vesicles derived from cells by optical tweezer. Sequence of cartoons (A–C) and transmission optical micrographs (D–F) showing the production of a native vesicle (arrow) pulling off from the cell’s plasma membrane by an optical tweezer; here we used HEK cells expressing the A2AR-YFP (Video S1). (G) Fluorescence micrograph of the vesicle obtained in (F) exhibiting fluorescence of the membrane expressed receptor. (H,I) Transmission optical micrographs showing the isolation of a native vesicle (arrow) selected by an optical tweezer from HEK cells expressing arrestin-GFP and NK1R. (J,K) Fluorescence micrographs of the vesicle before (J) and after (K) perfusion with the NK1R agonist substance P showing enhanced concentration of arrestin-GFP at the plasma membrane; insets are the corresponding fluorescence profile cross-sections of the vesicles.

Our results demonstrate that cell-derived vesicles act as autonomous functional containers capable to perform signaling reactions as their mother cells. Up until here, experiments were performed on vesicles derived from cytochalasin B treated cells. This procedure might deliver a vesicle population of heterogeneous protein expression, primarily reflecting the heterogeneity of individual cells within a population. Increasing evidence on the biochemical and functional variability between individuals in a population of cells demonstrate the importance of single-cell analysis for elucidating the cell’s function, especially in the context of the development and treatment of diseases [15]. In the following, we show that with the help of an optical tweezer [28] several vesicles can be drawn from a single live cell, where each vesicle is capable to perform signaling reactions thus multiplexing single-cell analysis. When an optical tweezer is focused on the cell surface, a part of the plasma membrane is trapped in the focus. By drawing away this membrane patch, a membrane nanotube is formed, which eventually ruptures off forming a closed vesicle containing a part of the cytosol and the plasma membrane of the mother cell (Figure 5D–G, Video S1). This form of trapping is enabled by the refractive index difference between the bulk medium and the cell. Whether the plasma membrane alone or together with the cytoplasm is finally responsible for the trapping is not clear yet. It has been shown elsewhere that a pure lipid vesicle with identical inner and outer solutions could be trapped and distorted in a laser focus due to optical forces acting on the lipid bilayer [29]. The high refractive index of a plasma membrane [29] therefore might be sufficient for trapping and pulling. High concentration of intracellular proteins and also the presence of submicrometer intracellular particles could reinforce the optical trapping/pulling process.

Preformed plasma membrane blebs induced by cytochalasin B serve as store of vesicles on the surface of a single cell from where they can be easily removed individually (Figure 5H,I) and placed at a defined position on the glass surface using an optical tweezer. Such vesicles retain the functional integrity of the GPCR signaling cascade. This is demonstrated for a vesicle containing the NK1R and arrestin-GFP: when the NK1R was activated by substance P, arrestin redistributed from the cytosol to the vesicle’s plasma membrane (Figure 5J,K).

### Conclusion

Taken together, we have demonstrated that individual plasma membrane vesicles function as minimized autonomous containers, capable of detecting external chemical signals and transducing them across the membrane to finally activate downstream signaling reactions inside the sub-femtoliter closed volume. These vesicles offer a generic platform for bioanalysis of transmembrane signaling going beyond presently reached miniaturization and flexibility with many potential future applications: (i) Vesicles contain a constant number of lipids and proteins allowing with the help of single-molecule and super-resolution microscopies [30], [31] quantification of the distribution and interaction of cellular signaling components at a precision that presently cannot be met in live cells. This would provide a dynamic tomogram of the complex cellular signaling network and thus provide substantial new insight to the basics of cellular function. (ii) The integration of individual vesicles in multi-arrays could reach an unprecedented high-density of biological functions enabling a massive increase of throughput and reduction of reagent consumption for functional screening of compounds or receptors [4], [6]. Examples are applications in fundamental bio-medical research to screen the function large numbers of receptors (e.g. naturally occurring subtypes or disease related mutants) when exposed to potential active compounds; this would be of direct importance for practical screening applications in pharma- (drug developments) and food- (functional food development) industry or for environmental monitoring of harmful substances. (iii) Since a multitude of vesicles can be derived from a single cell either in a spatially uncontrolled manner using cytochalasin [3] or in form of a locally controlled single vesicle biopsy, they would be ideally suited for multiplexing single-cell analysis to study heterogeneity between individual cells as well as within one cell at different states of cell development. This might be of interest for investigating the diversity of rare primary cells for example tumor or stem cells with direct impact to novel personalized cancer therapies [32]–[34].

## Materials and Methods

### Materials

Cytochalasin B and poly-D-lysine were purchased from Sigma-Aldrich (Buchs). Dulbecco’s modified Eagle’s medium (DMEM), fetal calf serum, Dulbecco’s phosphate-buffered saline (D-PBS) and AlexaFluor 647 were from Invitrogen. Human adenosine 2A receptor fused C-terminally to EYFP, human Gγ_2_ fused N-terminally to ECFP and human β-arrestin2 fused N-terminally to EGFP were constructed as described previously [24], [35], [36].

### Cell Culture and Transfection

For all experiments we used HEK293T cells cultured in T25 flask, grown in DMEM/F-12+ GlutaMAX medium containing 10% fetal calf serum in a humified atmosphere with 5% CO_2_ at 37°C. Cells were transiently transfected by Effectene (Qiagen) according to the manufacturer’s instruction. Ligand binding to GPCRs was measured on cells expressing adenosine 2A receptor fused at the C-terminus to YFP (A2AR-YFP). G-protein activation was measured in cells expressing A2AR-YFP, Gα_s_, Gβ_1_ and fluorescent Gγ_2_-CFP. Arrestin recruitment was monitored in cells that were transfected with fluorescent β-arrestin2-GFP and neurokinin 1 receptor (NK1R).

### Cell-Derived Plasma Membrane Vesicles Production

Cells were seeded in T25 flasks and cultivated to 80% confluence. After 18 hours cultivation cells were collected in PBS buffer containing 5 mM EDTA, centrifuged and resuspended in 5 ml D-PBS containing 25 µg/ml cytochalasin B. Native vesicles were sheared off from cells during 10 minutes of agitation at 700 rpm (IKA MS1 Minishaker), separated from cells by filtration (2 µm TPPC filter, Millipore), collected by centrifugation at 6,300 g for 10 min and finally resuspended in D-PBS buffer.

### Synthesis of XAC-Atto655

XAC-Atto655 was synthesized using XAC (Sigma) and Atto655-NHS (Atto-tec) as described elsewhere [24]. The identity of the HPLC purified product was confirmed by mass spectrometry (m/z = 938.6 (M^+^H^+^)).

### FRET Imaging and Analysis

Native vesicles were seeded on 8-well coverglasses (Nunc) coated with poly-D-lysine. FRET measurements were performed using an Axiovert 200 M inverted microscpe equipped with an EC Plan-NEOFLUAR 40×/1.3NA oil immersion objective lens (Zeiss). A XBO 75 W/2 xenon short-arc lamp (Zeiss) was used as a light source; images were recorded with an ORCA-ER digital camera (Hamamatsu) controlled by the IQ software (Andor). For dual wavelength imaging a beam splitter (Cairn Research) was used. FRET was measured as the ratio of the fluorescence intensities of YFP over CFP by exciting at 436±10 nm and monitoring the fluorescence at 465±15 nm (CFP) and 545±20 nm (YFP). Examined vesicles were carefully chosen to ensure that fluorescence levels of Gγ_2_-CFP and A2AR-YFP were comparable. The molar ratio of Gγ_2_-CFP to A2AR-YFP was estimated to be 0.96±0.37 (n = 20) as measured by FCS.

### Confocal Fluorescence Microscopy

The binding of fluorescent ligands to the vesicle surface and the recruitment of fluorescent arrestin were measured by confocal fluorescence microscopy. Plasma membrane vesicles were seeded on 8-well coverglasses (Nunc) coated with poly-D-lysine. The vesicles were imaged with a LSM510 confocal microscope equipped with a C-Apochromat 63×/1.2NA water immersion objective (Zeiss).

### Fluorescence Correlation Spectroscopy

FCS measurements were performed using a LSM 510 Meta laser scanning microscope based on an Axiovert 200 M microscope stand and equipped with a ConfoCor 3 FCS unit (Zeiss). The setup allowed acquisition of photon-count time traces and online correlation of the data. A2AR-YFP and XAC-Atto655 were excited at 514 nm (Ar-ion laser) and 633 nm (He-Ne laser), respectively. Fluorescence from A2AR-YFP and XAC-Atto655 was collected through a BP530–500 and a LP650 emission filter, respectively. An acousto-optical filter (AOTF) was used to adjust the exciting beam from the microscope objective (40× C-Apochromat, 1.2NA, water immersion, Zeiss) below 2 kW/cm^2^ for the Ar-ion laser and 10 kW/cm^2^ for the He-Ne laser to minimize photobleaching and photophysical effects. The lateral beam waist radius ω_0_ of the focused Ar-ion and He-Ne lasers was determined by measuring the translational diffusion time *τ_D_* of purified YFP (*D* = 92.3 µm^2^/s) [37] and Alexa Fluor 647 (*D* = 330 µm^2^/s) [38] according to *ω_0_* = (4*Dτ_D_*)^1/2^, and the respective detection volumes with *V_eff_* = *S*(4*πτ_D_D*)^3/2^.

Receptor diffusion and ligand binding were measured on native vesicles immobilized on a poly-D-lysine coated 8-well coverglasses (Nunc). To address the apical membrane of a particular vesicle, the confocal observation volume was first positioned in the center of the vesicle followed by an upward fluorescence intensity z-scan. The maximum of the fluorescence intensity in the z-scan indicates the position of the apical membrane. The confocal observation volume was adjusted above this position reaching half of the maximal intensity. Fluorescence intensity time traces and correlation curves were recorded for ten times 10 seconds. The traces of the autocorrelation function (ACF) were fitted using a Marquardt algorithm with Igor Pro (WaveMetrics, Lake Oswego, OR). ACFs of receptor diffusion were evaluated applying a single-component 2D model including triplet state formation:
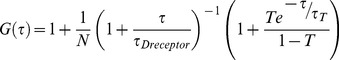
where *N* is the total number of receptors in the observation volume, *τ_Dreceptor_* is the translational diffusion time of the receptor, *T* and *τ_T_* are respectively the fraction and time constant of the triplet state formed.

For determining ligand binding, the ACF of XAC-Atto655 was fitted as follows combining the three-dimensional diffusion of free ligands in solution and the two-dimensional diffusion of bound ligands:
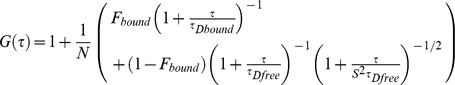
where *N* is the total number of ligands in the observation volume, *τ_Dbound_* and *τ_Dfree_* are the diffusion times of the bound and free ligand, respectively, *S* is the structure parameter defined by the ratio of the axial and lateral axes of the observation volume and *F_bound_* is the fraction of bound ligand.

ACFs of freely diffusing molecules were fitted with a three-dimensional diffusion model including triplet formation:
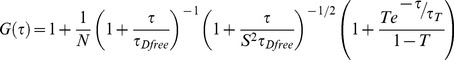



For ligand binding experiments, vesicles were incubated for 30 min with various concentrations of XAC-Atto655 and *K_D_* was determined by plotting the apparent concentration of bound ligand to the total concentration of applied ligand. Specificity of the binding was assessed by a subsequent addition of an excess of unlabeled XAC.

### Production of Vesicles by Optical Tweezers

The optical tweezer setup was built around an inverted microscope (Axiovert 200 M, Zeiss, Germany). For optical trapping, an Ytterbium fiber laser with linear polarization (PYL-10-1064- LP, IPG Photonics, USA) emitting up to 10 W cw at 1064 nm in a TEM_00_ mode was expanded by a telescope such that the beam slightly overfilled the back aperture of the microscope objective (Plan-Apochromat 100×/1.4 Oil, Zeiss, Germany). An additional solid-state laser emitting at 488 nm (FCD488, FDSU, USA) was available for fluorescence excitation. After passing a laser shutter the laser beam was expanded, coupled into the optical path of the IR laser by means of a dichroic mirror (Chroma, USA) and focused on the back focal plane of the microscope objective. Infrared trapping and fluorescence excitation laser light beams were separated from the emitted fluorescence light using a dielectric mirror (Chroma, USA) and appropriate filters. A CCD camera (Pixelfly, PCO, Germany) was used for fluorescence and brightfield imaging. Samples were clamped on a joystick-controlled motorized scanning stage. Membrane protrusions of blebbing cells, either drawn by the optical tweezer or formed under influence of cytochalasin B, were in both cases teared off from the cells by the optical tweezer during translation of the scanning stage. To be able to completely remove vesicles from the cells, a laser power of up to 2 W had to be employed. For analysis, vesicles were then deposited on the cover slide and the optical trap shut off.

### Statistical Analysis

Data are shown as means±standard deviations of the mean for *n* observations.

## Supporting Information

Figure S1 **Plasma membrane vesicle diameter.** Histogram of plasma membrane vesicle diameter distribution (threshold fixed at 500 nm).
(TIF)

Figure S2 **FCS analysis of A2AR-YFP in cells.** (A) Cartoon illustrating the position of the detection volume that was placed at the apical membrane over the nucleus for measuring receptor-bound-ligand or elsewhere in solution for measuring the free ligand. (B) Receptor diffusion. Autocorrelation curves (grey) fit by a single-component 2D diffusion model and considering triplet state formation (black): in this example the diffusion time of the receptor correspond to τ*_D_*_R_ = 64±22 ms (*D* = 0.21 µm^2^/s) and the chromophore blinking time is τ*_T_* = 507±303 µs. (C) Ligand binding. Normalized autocorrelation curves of XAC-Atto655 measured in the supernatant (blue) and at the apical membrane of the cell (black). (D) Receptor binding of XAC-Atto655 at different concentrations yielded a dissociation constant of *K_D_* = 97±12 nM. Shown are data points and standard error of the mean of three independent titrations performed on different individual cells.
(TIF)

Figure S3 **Ligand binding to A2AR-YFP measured in plasma membrane vesicles.** Normalized autocorrelation curves of XAC-Atto655 measured in the supernatant (blue), at the apical membrane of the vesicle (red) and after competition with non-fluorescent XAC (green).
(TIF)

Figure S4 **Analysis of receptor/G protein interaction by FRET in cells.** Confocal micrographs of HEK cells expressing Gγ_2_-CFP (A) and A2AR-YFP (B); scale bar: 10 µm. (C) FRET changes in single cell in response to increasing concentrations of agonist APEC. (D) Concentration–response curves of receptor/G protein interaction yields *EC_50_* = 76±29 nM (n = 5). Shown are data points and standard error of the mean of five independent titrations performed on different individual cells.
(TIF)

Figure S5 **Analysis of receptor/G protein interaction by FRET in vesicles.** Confocal micrographs of plasma membrane vesicles derived from HEK cells expressing heterologously (A) Gγ_2_-CFP and (B) A2AR-YFP; scale bar: 10 µm. (C–F) FRET changes in single vesicles in response to increasing concentrations of agonist APEC.
(TIF)

Video S1 **Production of a vesicle by an optical tweezer.** Movie showing the production of a single plasma membrane vesicle pulled off from a cell’s plasma membrane by an optical tweezer.
(MOV)
