# Supplementary figures and images for: Downscaling the Analysis of Complex Transmembrane Signaling Cascades to Closed Attoliter Volumes
Source: PLoS One. 2013 Aug 5;8(8):e70929. doi: 10.1371/journal.pone.0070929 (PMC3733713; doi:10.1371/journal.pone.0070929)

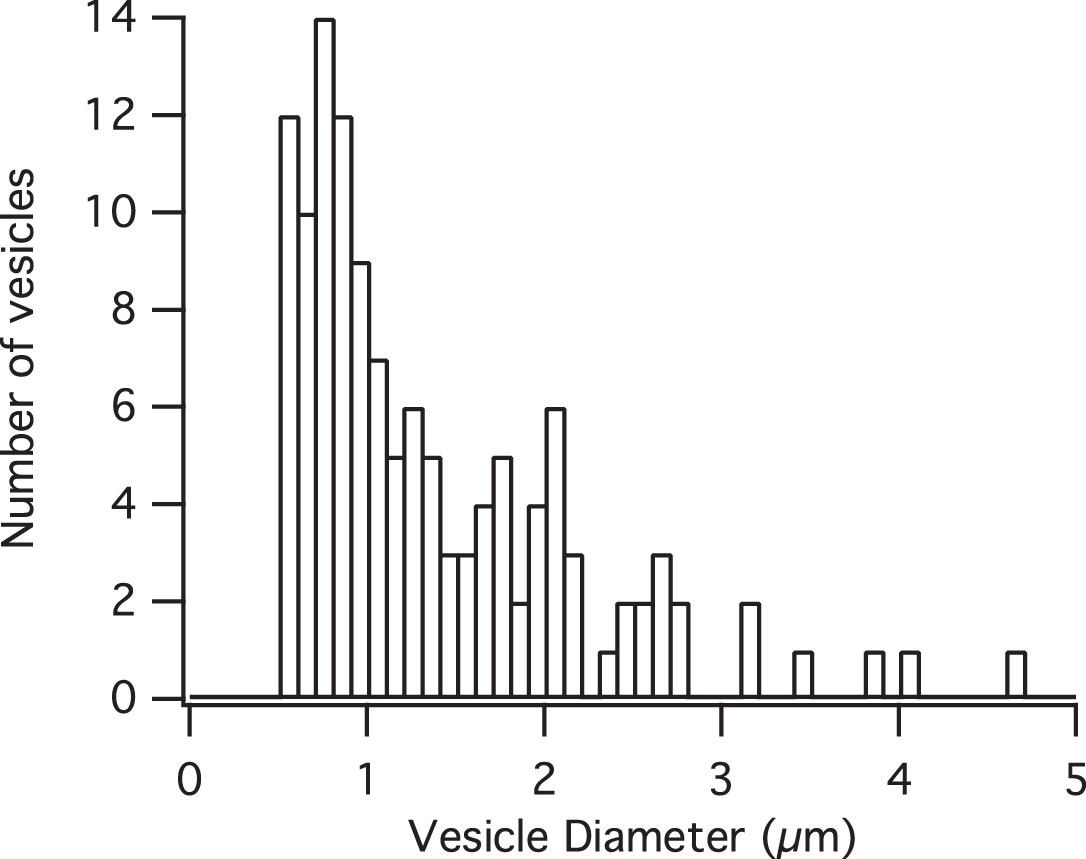

Supplement: Figure S1 — Plasma membrane vesicle diameter. Histogram of plasma membrane vesicle diameter distribution (threshold fixed at 500 nm). (TIF) [file pone.0070929.s001.tif]

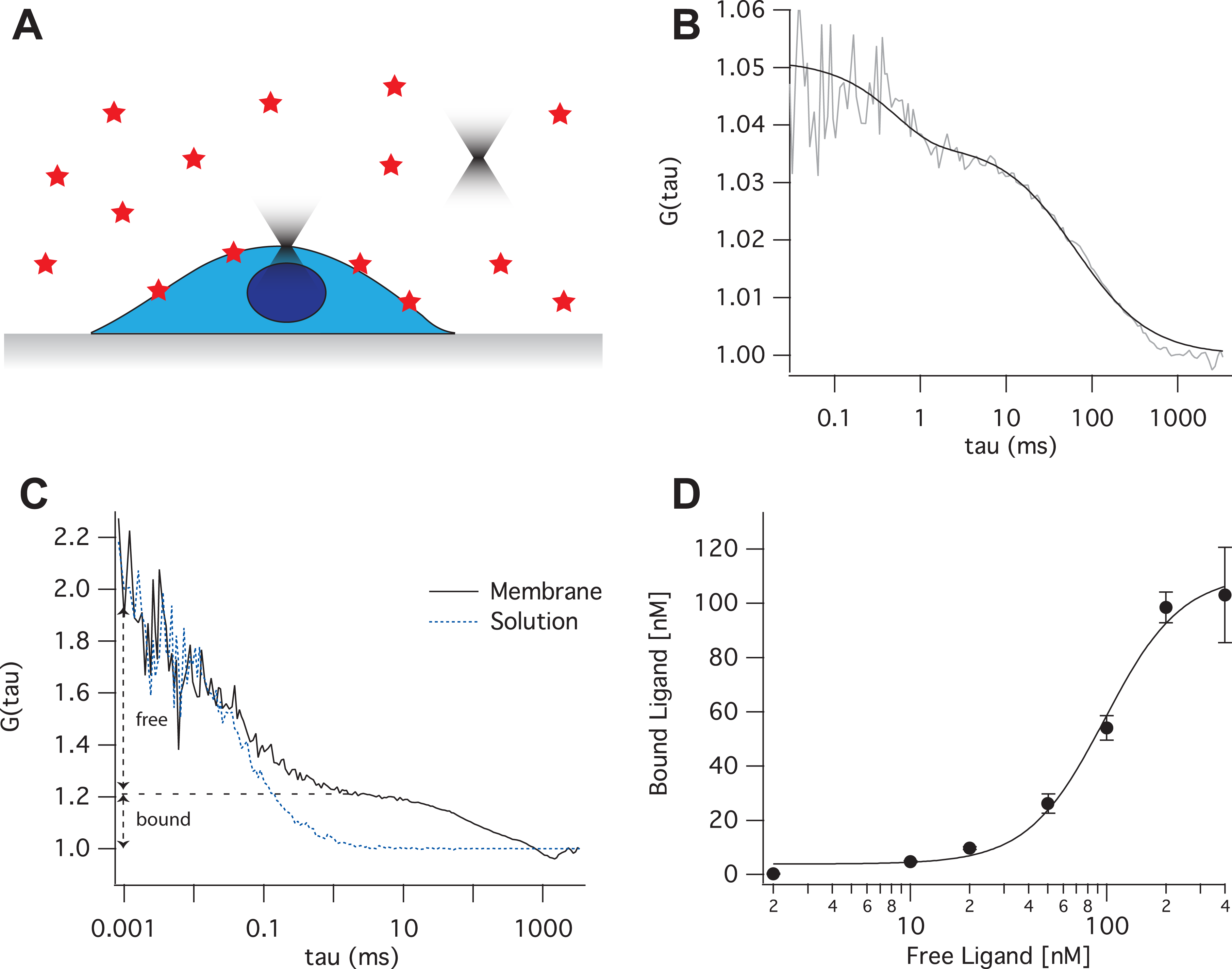

Supplement: Figure S2 — FCS analysis of A2AR-YFP in cells. (A) Cartoon illustrating the position of the detection volume that was placed at the apical membrane over the nucleus for measuring receptor-bound-ligand or elsewhere in solution for measuring the free ligand. (B) Receptor diffusion. Autocorrelation curves (grey) fit by a single-component 2D diffusion model and considering triplet state formation (black): in this example the diffusion time of the receptor correspond to τD R = 64±22 ms (D = 0.21 µm2/s) and the chromophore blinking time is τT = 507±303 µs. (C) Ligand binding. Normalized autocorrelation curves of XAC-Atto655 measured in the supernatant (blue) and at the apical membrane of the cell (black). (D) Receptor binding of XAC-Atto655 at different concentrations yielded a dissociation constant of KD = 97±12 nM. Shown are data points and standard error of the mean of three independent titrations performed on different individual cells. (TIF) [file pone.0070929.s002.tif]

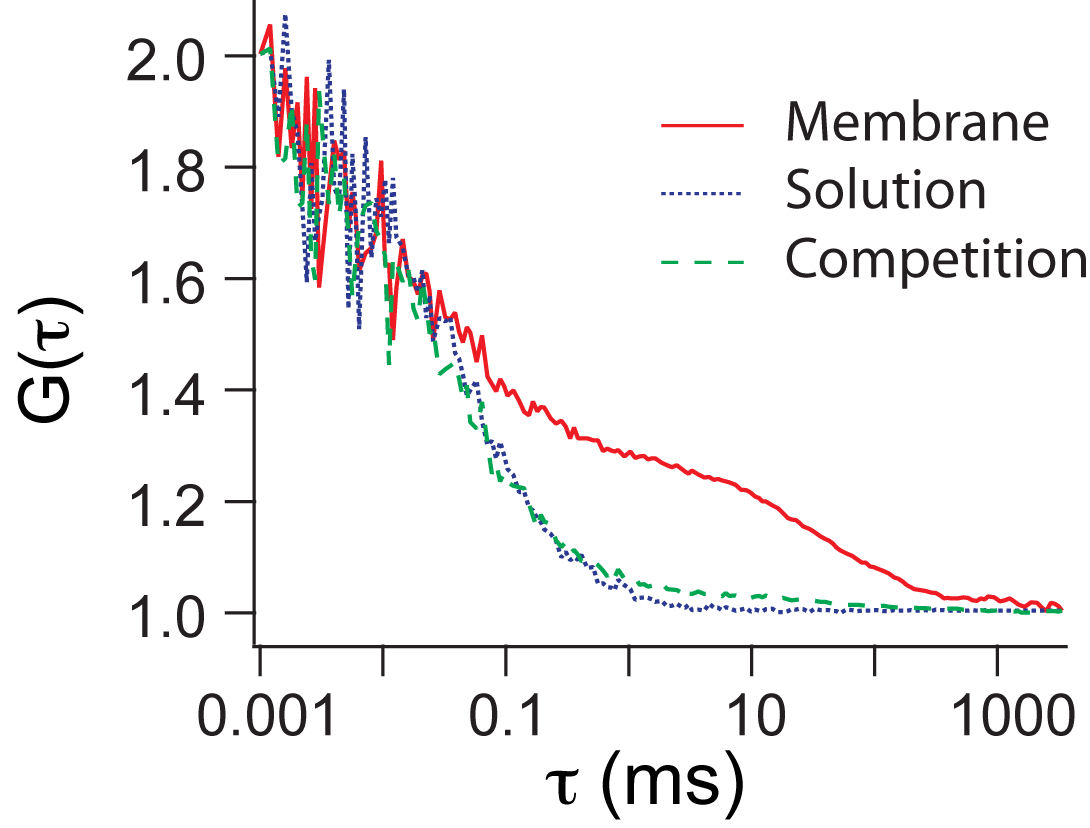

Supplement: Figure S3 — Ligand binding to A2AR-YFP measured in plasma membrane vesicles. Normalized autocorrelation curves of XAC-Atto655 measured in the supernatant (blue), at the apical membrane of the vesicle (red) and after competition with non-fluorescent XAC (green). (TIF) [file pone.0070929.s003.tif]

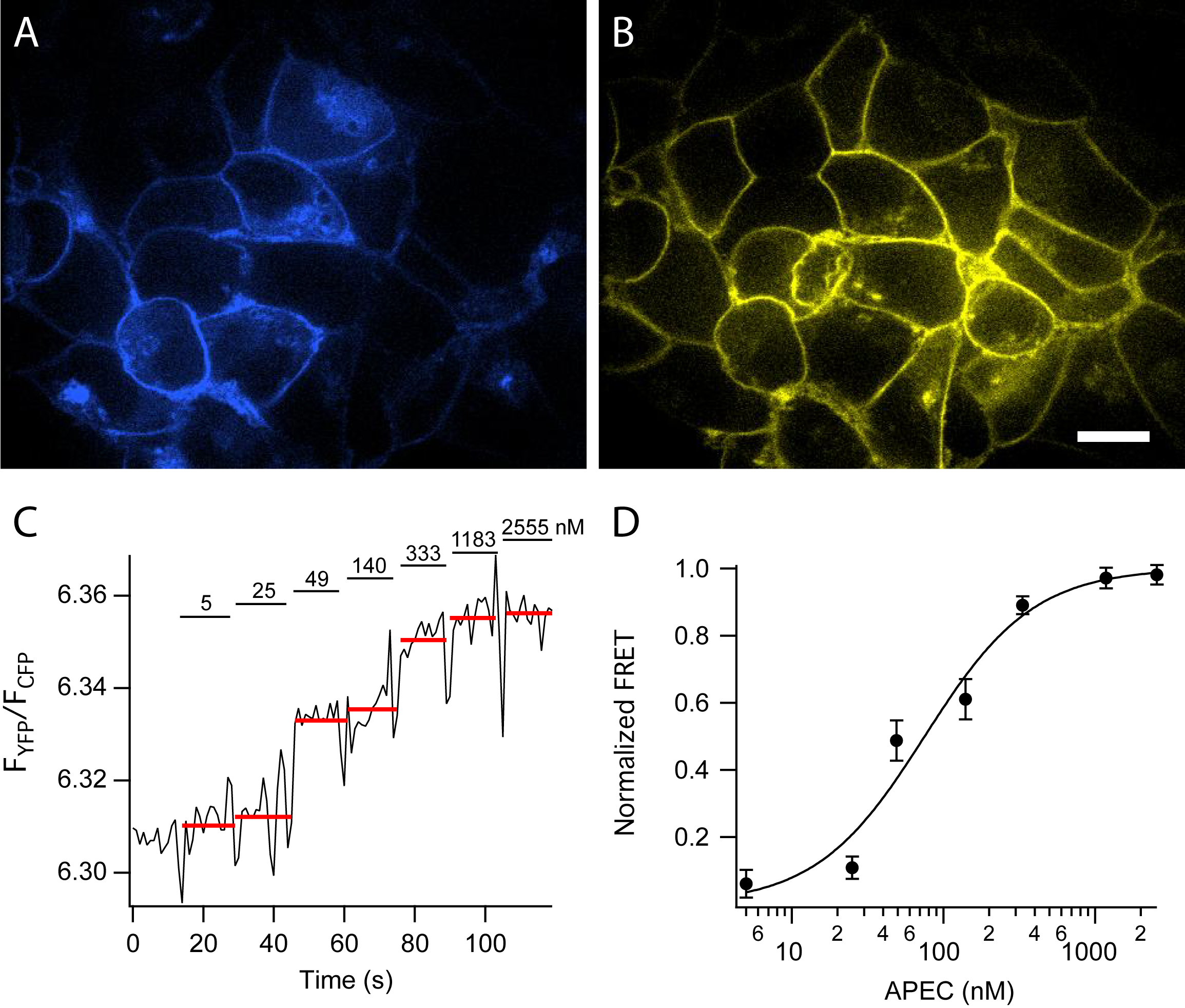

Supplement: Figure S4 — Analysis of receptor/G protein interaction by FRET in cells. Confocal micrographs of HEK cells expressing Gγ2-CFP (A) and A2AR-YFP (B); scale bar: 10 µm. (C) FRET changes in single cell in response to increasing concentrations of agonist APEC. (D) Concentration–response curves of receptor/G protein interaction yields EC50 = 76±29 nM (n = 5). Shown are data points and standard error of the mean of five independent titrations performed on different individual cells. (TIF) [file pone.0070929.s004.tif]

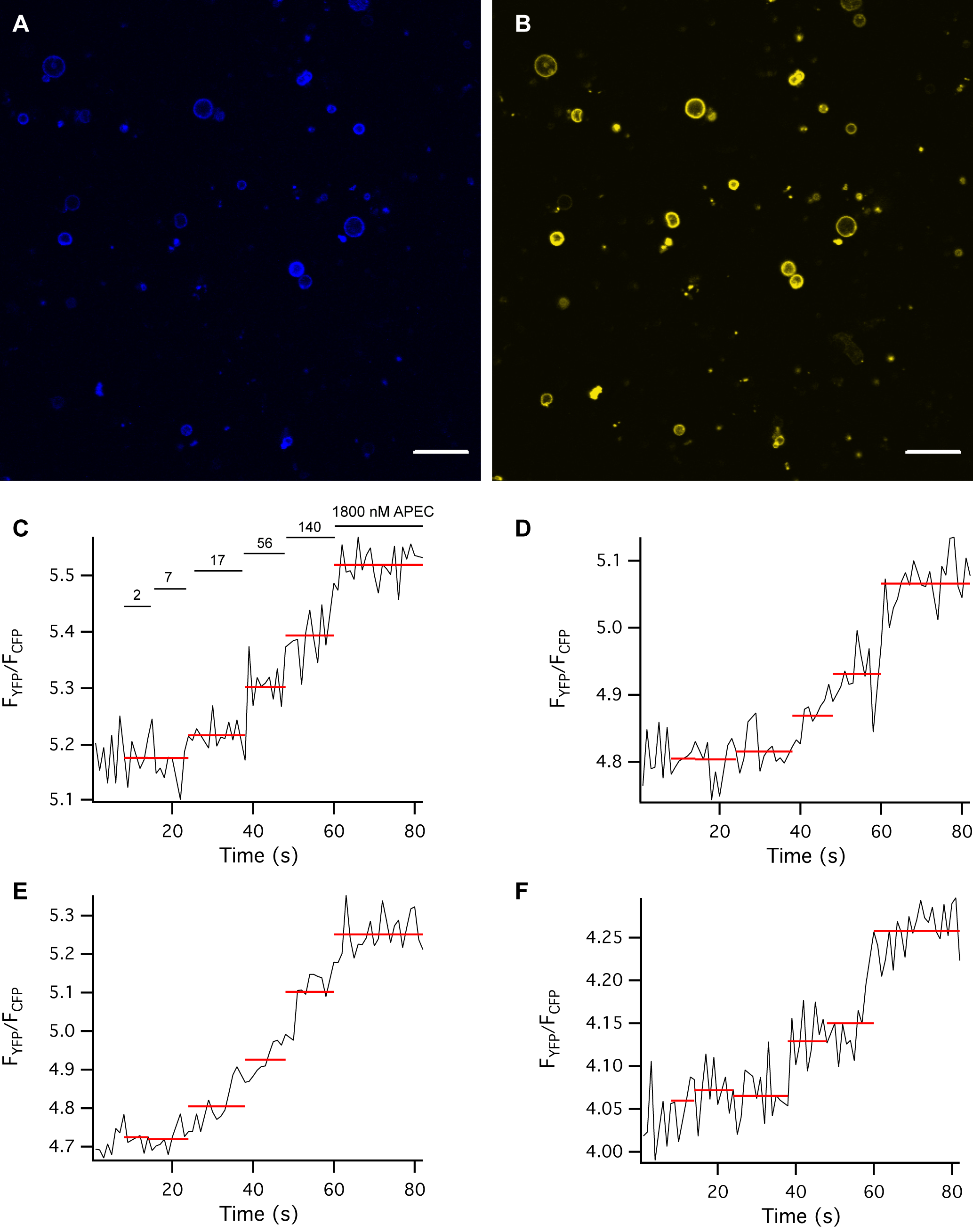

Supplement: Figure S5 — Analysis of receptor/G protein interaction by FRET in vesicles. Confocal micrographs of plasma membrane vesicles derived from HEK cells expressing heterologously (A) Gγ2-CFP and (B) A2AR-YFP; scale bar: 10 µm. (C–F) FRET changes in single vesicles in response to increasing concentrations of agonist APEC. (TIF) [file pone.0070929.s005.tif]
